# Annual Risk of Tuberculous Infection Using Different Methods in Communities with a High Prevalence of TB and HIV in Zambia and South Africa

**DOI:** 10.1371/journal.pone.0007749

**Published:** 2009-11-13

**Authors:** Kwame Shanaube, Charalambos Sismanidis, Helen Ayles, Nulda Beyers, Ab Schaap, Katherine-Anne Lawrence, Annie Barker, Peter Godfrey-Faussett

**Affiliations:** 1 ZAMBART Project, University Teaching Hospital, Lusaka, Zambia; 2 London School of Hygiene and Tropical Medicine, London, United Kingdom; 3 Desmond Tutu TB Centre, Stellenbosch University, South Africa; McGill University, Canada

## Abstract

**Background:**

The annual risk of tuberculous infection (ARTI) is a key epidemiological indicator of the extent of transmission in a community. Several methods have been suggested to estimate the prevalence of tuberculous infection using tuberculin skin test data. This paper explores the implications of using different methods to estimate prevalence of infection and ARTI. The effect of BCG vaccination on these estimates is also investigated.

**Methodology/Principal Findings:**

Tuberculin surveys among school children in 16 communities in Zambia and 8 in South Africa (SA) were performed in 2005, as part of baseline data collection and for randomisation purposes of the ZAMSTAR study. Infection prevalence and ARTI estimates were calculated using five methods: different cut-offs with or without adjustments for sensitivity, the mirror method, and mixture analysis. A total of 49,835 children were registered for the surveys, of which 25,048 (50%) had skin tests done and 22,563 (90%) of those tested were read. Infection prevalence was higher in the combined SA than Zambian communities. The mirror method resulted in the least difference of 7.8%, whereas that estimated by the cut-off methods varied from 12.2% to 17.3%. The ARTI in the Zambian and SA communities was between 0.8% and 2.8% and 2.5% and 4.2% respectively, depending on the method used. In the SA communities, the ARTI was higher among the younger children. BCG vaccination had little effect on these estimates.

**Conclusions/Significance:**

ARTI estimates are dependent on the calculation method used. All methods agreed that there were substantial differences in infection prevalence across the communities, with higher rates in SA. Although TB notification rates have increased over the past decades, the difference in cumulative exposure between younger and older children is less dramatic and a rise in risk of infection in parallel with the estimated incidence of active tuberculosis cannot be excluded.

## Introduction

The annual risk of tuberculous infection (ARTI) is an epidemiological index derived from tuberculin skin test (TST) surveys among children to measure the extent of TB transmission in a community. It is the probability of acquiring new tuberculous infection or reinfection over a period of one year. ARTI trends are a critical indicator for progress, or lack thereof, in tuberculosis control in a community. In the recent past, ARTI estimates were used by TB control programmes to estimate the incidence of smear positive TB at a population level by using the Styblo rule [1]. However, it is now widely accepted that this assumed fixed mathematical relationship between ARTI and the incidence of TB is no longer valid [2]. In addition, ARTI trends have been used to assess the impact of the HIV epidemic on TB transmission [3], [4] although it is still argued that the risk of infection in children allows little insight on the impact that HIV may exert on the burden of active tuberculosis in a population [5].

Despite the huge experience gathered over the past century, the operating characteristics of the TST ensure that there will always be a trade-off between sensitivity and specificity and that these will vary with the prevalence of infection with *M. tuberculosis* and other mycobacteria, including Bacille Calmette-Guerin (BCG) in the population. The predictive value of the test will also depend on the prevalence of tuberculous infection in the particular population [6]. Recent discussion has focused on the use of new technologies to determine who is infected [7] and on more sophisticated statistical methodologies in the interpretation of TST data to estimate the infection prevalence [8]–[10].

The most straightforward method is to use a predetermined cut-off value above which individuals are presumed to be infected with *M.tuberculosis*. The value proposed for the cut-off may take into account the prior probability of being infected or the immune status of the individual [11]. A less arbitrary variant on this approach defines the cut-off in order to produce a high specificity among an unexposed population and then increases the calculated prevalence of infection by a factor derived from the observed sensitivity of the chosen cut-off in a population of patients with proven tuberculosis, who must therefore already have tuberculous infection [12].

Mirror methods are used to define prevalence in a population and do not assign all individuals to infected or non-infected states. The principal assumption is that the frequency distribution of indurations caused by tuberculous infection will be symmetrically distributed. The first approach assumes that the mode of the distribution reflects infection due to *M.tuberculosis* so that the prevalence of infection can be calculated by doubling the frequency of individuals whose induration is greater than the mode and adding those whose induration equals the mode [13]. A related approach is to use a value derived from previous data on the distribution in different populations and to assume this value to be the mode of the frequency distribution attributable to *M.tuberculosis* regardless of the observed distribution in the population [14].

Most recently, mathematical modelling approaches have been used which examine the likelihood of different theoretical populations being mixed together to produce the observed distribution [9], [15], [16]. Within such “mixture methods”, it is then assumed that one population reflects infection, while one (or more) populations reflect non-specific reactions to BCG, environmental mycobacteria or background noise in the test's operating characteristics. There is also currently growing interest in another type of mixture model, called a latent class model, for analysing the results of multiple dichotomised tests [10].

We present data from two tuberculin skin test surveys conducted in 2005 as baseline studies of a large community randomised trial [17], [18] called ZAMSTAR (Zambia South Africa TB and AIDS Reduction). The ZAMSTAR study evaluates two public health interventions that aim to reduce the prevalence of TB at community level across 16 communities in Zambia and 8 communities in the Western Cape Province of South Africa (SA). These tuberculin surveys served three objectives: to characterize ZAMSTAR communities with regards to tuberculous infection; to inform the randomization of the communities into the four intervention arms [18]; and to provide data for one of ZAMSTAR's secondary outcomes.

The primary objective of this paper is to estimate the prevalence of tuberculous infection and ARTI among school children aged 6–11 years in 24 communities in Zambia and South Africa using five methods. We also explore whether these methods alter the ranking of our communities, with regards to tuberculous infection. Finally, we examine the effect of BCG vaccination on prevalence of infection estimates.

## Materials and Methods

### Ethics statement

Written consent for tuberculin testing was obtained from the parent or guardian of every child. The study protocol was approved by the Ethics committees of the University of Zambia and Stellenbosch University as well as the London School of Hygiene and Tropical Medicine.

### Study setting

The study was conducted in 24 selected communities in Zambia and Western Cape, South Africa. The term “community” (unit of randomisation) was defined as the population (minimum size of 25,000) accessing one TB diagnostic centre. Study communities were selected based on TB notification rates greater than 400/100,000 per annum, high HIV seroprevalence and proximity to a TB diagnostic centre. The communities selected were in five provinces of Zambia and in Western Cape Province of South Africa and included both urban and rural communities. Multi-stage purposive sampling for choosing communities was used.

### ZAMSTAR study community ranking

The details of the design and randomization of the ZAMSTAR study are described elsewhere [17], [18]. However, briefly for the randomization of the ZAMSTAR trial, we used stratification and restriction to randomize 24 clusters into four intervention arms in a 2×2 factorial design. To ensure that intervention effects were not distorted due to baseline imbalances between intervention groups, communities were ranked according to their TST prevalence estimates within country. Stratification was by country and tuberculous infection prevalence and restriction by tuberculous infection prevalence, HIV prevalence, urban/rural, social context, and geographical location.

### Survey design and sample size

The primary schools that served the children within the community and closest to the TB diagnostic centre were selected. The TST surveys were conducted in 98 schools (56 in Zambia and 42 in SA), in the 24 communities. Our target sample size was 800 children aged between 5–9 years (grade 1–3) per community (19,200 in total). This target sample size was based on estimations for one of ZAMSTAR study secondary outcomes and has been explained in detail elsewhere [17].

### Tuberculin skin testing

All children enrolled in grades 1 to 3 were eligible for inclusion in the survey. Survey staff were trained in the placement and reading of tuberculin skin tests according to the standard IUATLD protocol [19]. Training included exchange visits between Zambia and SA so that the trainers were using the same methods throughout. To standardise the reading of the tests healthy volunteers and TB patients were used. In SA, one team conducted the survey in all the 8 communities, while in Zambia, 6 teams located in the different geographical areas conducted the survey. Permission was obtained from the Ethics committees, departments of health and education, school authorities and community leaders.

Children were listed with their age, sex and address on a data collection form based on the school register. The size of the induration and the presence of a BCG scar as verified by a nurse were also recorded. Children were included in the survey whether they had a BCG scar or not. The skin testing was conducted using 2TU (Tuberculin Units) of PPD RT23 with Tween, supplied by the Statens Serum Institut (Copenhagen, Denmark). A single batch was prepared for these surveys. A dose of 0.1 ml was injected intradermally on the left forearm. Skin reactions were read using callipers 72 hours later. All pupils with reactions of ≥15 mm were referred to the clinic to be investigated for TB disease. In keeping with national guidelines, as the children were older than 5 years, they were not referred for prophylaxis. Data was dually entered.

### Different methods to estimate prevalence of infection

Histograms of induration sizes were inspected for evidence of digit-preference and multi-modality of distributions. Tuberculous infection prevalence was calculated as the proportion of all children with a TST positive result over the total number of children with an administered and read skin test. TST positivity was defined using cut-off, mirror and mixture analysis methods suggested by the literature, ranging from the simple cut-off value [12], [14], [20] approaches to the more sophisticated mixture analysis [9], [15], [16]. For randomization of the ZAMSTAR study, we determined the balance between the study arms using each method. Our sample size allowed us to dissect the data, when using mixture analysis, at the country but not the community level, because larger numbers of non-zero observations were required for the models to converge. With all other approaches, we investigated variation of infection prevalence at the community level. Tuberculous infection estimates at the country level were calculated as unweighted averages of community level estimates using the cut-off and mirror methods. In addition, 95% confidence intervals were calculated in order to account for the clustering effect at community level [21].

#### Fixed cut-off points at 10 mm or 15 mm

Infection prevalence was calculated using 10 mm and 15 mm cut-off points. The 10 mm criterion is the most widely used that considers all reactions ≥10 mm to be a marker of infection [20], [22]. The 15 mm cut-off point has also been used elsewhere [20], [23]. To adjust for sensitivity, a cut-off of 14 mm was used and the number multiplied by 1.22 to correct for false negatives [12], [14]. The factor 1.22 has been established by data from population of infected individuals in Tanzania [12].

#### Mirror method

The mirror method was used to estimate prevalence using the mode as a mirror and a fixed mirror at 17 mm. The mode of the TST distribution was identified after smoothing (to adjust for obvious digit-preference bias) the crude count of TST indurations by a centred moving average of five successive reaction sizes. The total number of children with true reactions was calculated by adding the number of children showing reaction sizes equal to the mode to double the number with reaction sizes larger than the mode to determine the numerator [13], [15]. The fixed mirror method considers all reactions of 17 mm counted once and indurations of ≥17 mm counted twice to obtain the estimated number of infections [12], [14], [24].

#### Mixture analysis

Three parametric models (normal, lognormal and Weibull distributions) describing infection with M. tuberculosis, and two (lognormal and Weibull distributions) describing those who reacted due to infection with environmental mycobacteria were tested to determine the best model.

A Bayesian Markov Chain Monte Carlo simulation approach [25] and programme codes for the R software were utilised for this analysis. The Metropolis-Gibbs sampler was used to calculate posterior distribution of mixture model parameters. The simulation programme initially ran for a burn-in period of 15,000 iterations the results of which were discarded. Following the burn-in period a thinned sample of 2,000 from 20,000 was used to summarise the posterior distribution of the model parameters. The validity of the model was assessable by how well it fitted the data. Models with maximum log likelihood values were used to quantify the fit. Comparisons between predicted and observed frequencies via posterior predictive model checks solidified the choice of model. For a model to be consistent with data, the posterior predictive failure rate was close to 5%. Tuberculous infection estimates at the country level were presented along with 95% confidence intervals unadjusted for the clustering effect of community level, as this was not possible using this method.

### Estimating the annual risk of tuberculous infection

We calculated the annual risk of infection from the prevalence of infection estimates. We used the standard formula R = 1−(1−Prevalence)^ 1/A +0.5^ where R is the probability of being infected in any one year and A is the mean age [8], [19]. Because the age (in full years) of each child at their last birthday was used, 0.5 was added to the mean age for the calculation of ARTI. Furthermore, two critical assumptions in the ARTI calculation were made. Firstly, we assumed that the ARTI was independent of the age of the person at risk of infection while exposure to TB is likely to change as people grow older. The second key assumption was that the ARTI was constant over time, which may not be the case. Because our surveys were done in children aged about 6 to11 years we estimated ARTI for each of the groups of 6, 7, 8, 9, 10 and 11-year-olds.

### Impact of age and BCG

Indirectly standardised prevalence estimates using the total school children population whose consent was sought as the standard population, were calculated using the formula below:
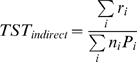



+

Where

*r_i_* is the number of children positive in the *i^th^* age group

*n_i_* is the number of children in the *i^th^* age group

*P_i_* is the proportion of children positive in the *i^th^* age group in the standard population

The age specific prevalence of infection were stratified by country only and estimated using all methods apart from the mirror method due to inadequate numbers. In the mixture analysis, age was included as a covariate in the models.

The infection prevalence was also compared between children with a BCG scar and those without using all methods (apart from mixture analysis) and age group. A mixture model was not used to compare children with a BCG scar and those without because the data restricted us from including an additional factor in the model.

## Results

### Survey participation

A total of 49,835 eligible children aged between 4–18 years were registered in the TST surveys. Of those registered 25,048 (50%) had skin tests done and 22,563 (90%) of children tested were read. There was little difference in mean age and sex distribution among children sought, administered and read (Table 1). Analysis was restricted to 6–11 year-olds (94% of children read) because frequencies in the youngest and oldest age groups were small. In Zambia, this age range corresponds to primary school (7–13 years) gross attendance rate of 98.2% and 87.2% in urban and rural areas respectively [26] while in Western Cape an overall of 97% [27].

**Table 1:** Children involved in the baseline tuberculin skin test surveys for all 24 ZAMSTAR communities.

Code	Geography	Consent sought N (% female) [mean age]	Administered N (% female) [mean age]	Read N (% female) [mean age]
Z1	Lusaka	2,914 (51) [9.4]	936 (56) [9.5]	806 (56) [9.5]
Z2	Copperbelt	2,287 (52) [8.2]	919 (53) [8.4]	903 (53) [8.4]
Z3	Copperbelt	3,011(52) [8.4]	1,145 (49) [8.2]	937 (49) [8.2]
Z4	Lusaka	2,901(50) [9.4]	956 (50) [9.5]	785 (51) [9.5]
Z5	Copperbelt	2,013(48) [9.5]	1,139 (47) [9.7]	927 (47) [9.7]
Z6	Lusaka	3,049(52) [9.6]	924 (52) [9.5]	806 (52) [9.5]
Z7	Lusaka	2,535(51) [9.2]	753 (51) [9.2]	704 (51) [9.2]
Z8	Southern	1,577(53) [7.9]	886 (55) [8.0]	788 (56) [8.0]
Z9	Southern	1,524(51) [8.0]	895 (51) [8.1]	759 (51) [8.1]
Z10	Central	2,171(51) [9.1]	1,286 (53) [9.1]	1,187 (53) [9.1]
Z11	Luapula	1,950(51) [8.8]	1,179 (52) [9.0]	1,064 (52) [9.0]
Z12	Copperbelt	2,115(51) [8.5]	936 (53) [8.6]	829 (54) [8.6]
Z13	Central	1,470(61) [8.3]	863 (63) [8.3]	830 (63) [8.3]
Z14	Southern	1,518(48) [8.7]	901 (48) [8.6]	872 (48) [8.6]
Z15	Luapula	1,663(51) [9.1]	933 (52) [9.2]	827 (52) [9.2]
Z16	Southern	1,732(50) [8.3]	949 (52) [8.2]	763 (53) [8.2]
SA1	Province	2,277(48) [7.5]	1,399 (49) [7.5]	1,319 (50) [7.5]
SA2	Province	1,678(46) [8.3]	913 (49) [8.2]	796 (47) [8.2]
SA3	Metropole	2,607(49) [8.1]	1,550 (50) [8.2]	1,438 (51) [8.2]
SA4	Metropole	1,626(48) [8.4]	1,028 (51) [8.3]	962 (51) [8.3]
SA5	Metropole	2,057(47) [7.5]	1,286 (48) [7.6]	1,232 (48) [7.6]
SA6	Province	890(48) [8.3]	561 (48) [8.4]	537 (48) [8.4]
SA7	Metropole	2,314(49) [8.2]	1,421 (49) [8.2]	1,292 (49) [8.2]
SA8	Metropole	1,956(46) [7.6]	1,290 (46) [7.6]	1,200 (46) [7.6]
**TOTAL**		**49,835 (50) [8.6]**	**25,048 (51) [8.5]**	**22,563 (51) [8.5]**

**Figure 1:**
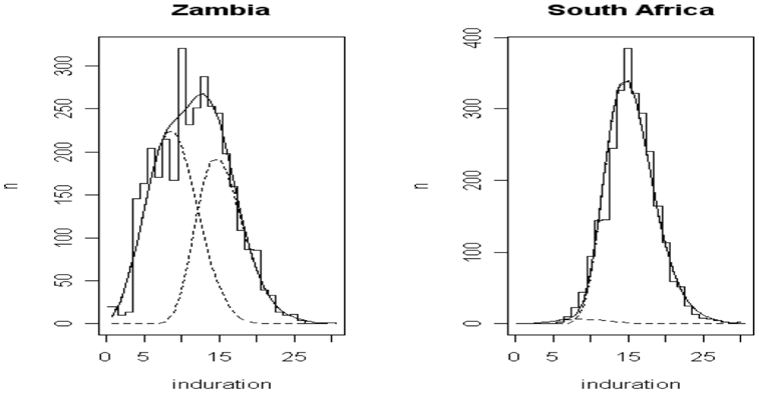
Histogram of frequencies of observed non-zero indurations as reactions to TST by country.

Despite the wide spread use of BCG, many children had no reaction to the TST (76% in Zambia and 69% SA). The frequency distribution of non-zero indurations is shown for each country (Figure 1). The Zambian distribution showed evidence of digit preference, but despite this, is still less symmetrical than the SA one, with a larger frequency of children with induration less than the mode than above it. The mode of the Zambian distribution, at 12 mm (discounting the 10 mm bin), was also 3 mm less than the SA 15 mm mode.

### Infection prevalence and ARTI estimates

Table 2 compares the infection prevalence estimates, at the community and country level, using all methods. For two of the communities with few non-zero readings, the frequency distribution did not have a single mode above 10 mm, so no value was calculated for the mirror method. Using these infection prevalence estimates, we calculated the ARTI for the Zambian communities to be as follows: 0.8% (15 mm); 2.0% (10 mm); 1.3% (14 mm*1.22); 2.8% (mirror); 0.8% (fixed mirror); 1.2% (mixture). The ARTI for the SA communities were as follows: 2.5% (15 mm); 4.2 % (10 mm); 3.8% (14 mm*1.22); 4.2% (mirror); 2.5% (fixed mirror); 4.2% (mixture).

**Table 2:** Tuberculous infection prevalence estimates by community and country.

		15 mm	15 mm	10 mm	10 mm	14 mm*1.22	Mirror	Fixed Mirror	Mixture
	Community code	*Crude*	*Adjusted* +	*Crude*	*Adjusted* +				
	Z16	1.8	1.8	6.0	6.1	3.0	N/A*	0.7	N/A*
	Z15	2.8	2.8	6.1	6.0	4.5	8.2	4.1	N/A*
	Z13	2.8	2.8	14.1	14.2	7.3	19.5	0.7	N/A*
	Z14	3.6	3.6	8.1	8.1	5.7	N/A*	3.5	N/A*
	Z12	3.9	3.9	14.9	14.9	6.7	18.1	4.0	N/A*
	Z10	4.8	4.8	19.3	19.2	8.7	25.2	2.1	N/A*
	Z11	5.1	5.1	9.6	9.5	6.3	10.3	4.3	N/A*
	Z9	6.4	6.5	16.9	17.2	10.3	21.9	4.7	N/A*
	Z8	7.6	7.6	17.3	17.5	11.4	20.8	6.4	N/A*
	Z3	8.5	8.6	19.8	20.0	13.4	24.3	7.9	N/A*
	Z2	9.7	9.7	21.3	21.3	13.8	29.5	10.0	N/A*
	Z1	10.8	10.7	22.1	21.7	16.0	33.2	11.5	N/A*
	Z5	11.4	11.3	23.9	23.4	15.9	31.5	13.3	N/A*
	Z6	11.9	11.8	21.5	21.0	17.1	22.6	17.0	N/A*
	Z7	12.0	12.0	23.8	23.4	17.6	26.5	5.7	N/A*
	Z4	13.0	12.9	21.9	21.4	18.8	24.2	15.6	N/A*
**Zambia (95%CI)**		**7.3 (5.4–9.1)**	**7.2 (5.5–9.0)**	**16.7 (11.9–21.4)**	**16.5 (12.0**–**21.1)**	**11.0 (7.8**–**14.3)**	**22.5 (15.5**–**29.6)**	**7.0 (3.8**–**10.2)**	**10.8 (9.1**–**12.1** * **)**
	SA8	14.2	14.3	26.0	26.5	21.6	24.8	10.8	N/A*
	SA7	14.7	14.8	24.8	24.9	22.1	18.6	13.8	N/A*
	SA5	17.0	17.0	28.5	29.0	24.9	23.8	18.1	N/A*
	SA6	17.1	17.2	26.3	26.3	23.9	31.8	18.7	N/A*
	SA2	18.7	18.7	31.7	31.8	29.6	32.8	17.0	N/A*
	SA3	22.3	22.4	32.0	32.1	32.4	29.4	19.9	N/A*
	SA1	22.6	22.7	30.4	31.1	30.8	33.8	27.4	N/A*
	SA4	27.1	27.1	42.4	42.6	39.0	47.8	27.9	N/A*
**South Africa (95%CI)**		**19.2 (14.4**–**24.0)**	**19.3 (14.4**–**24.1)**	**30.3 (22.6**–**38.0)**	**30.5 (22.9**–**38.2)**	**28.0 (19.2**–**36.9)**	**30.3 (11.6**–**49.1)**	**19.2 (10.5**–**28.9)**	**30.3 (29.4**–**31.1** * **)**

* Non-assessable.

+ Indirect age-standardized estimate using the total as the standard population

Country level estimates are calculated as unweighted averages of community estimates and 95% confidence intervals (CI) account for the clustering effect at the community level. Communities are ordered in ascending 15 mm cut-off method order.

For the mixture analysis method for the Zambian population, the best model fit was given by distributional assumption made for the observed cross-reaction/ tuberculous infection of Weibull/Lognormal and the prevalence was 10.8% ( 95% credible interval (CI): 9.1–12.1). For the SA population the Lognormal/Weibull and Normal/Weibull assumption both fit the model well and the prevalence was 30.3% (95% CI: 29.4–31.1).The distribution of observed (histogram), mixture distribution and component distribution of tuberculous infection and cross-reactions by country is shown in Figure 1. The mode of the distributions for presumed tuberculous infection given by the mixture analyses is 15 mm for both datasets.

**Figure 2:**
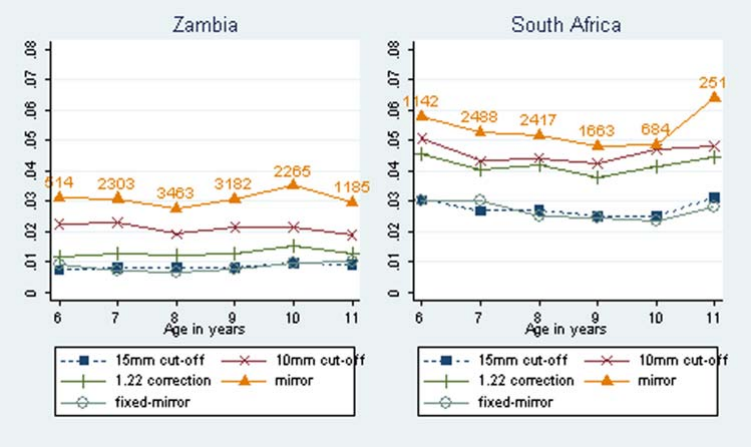
Annual risk of tuberculous infection, as calculated using five methods, by country and age.

For each method there was considerable variation in infection prevalence (Table 2) and ARTI (Figure 2) estimates among the communities and between countries. The mirror methods showed the highest and lowest infection prevalence estimates for Zambia. Infection prevalence estimates for the Zambian communities varied from 7.0% using the fixed mirror method to 22.5% using the mirror method. Similarly, for the SA communities, infection prevalence estimates varied from 19.2% using the fixed mirror method to 30.3% using mirror, mixture, or the 10 mm cut-off methods.

The differences in infection prevalence between the Zambian and SA communities varied from 7.8% using the mirror method to 19.5% using the mixture method. All methods suggested that the prevalence of infection was considerably higher in the SA communities than the Zambian ones (Table 2). However, with the methods using a cut-off, there was no overlap in the range of estimated prevalences, whereas when the mirror methods were used, many Zambian communities were estimated to have prevalences similar to those found in the SA communities (Table 2).

For the Zambian communities, there was little variation in the ARTI estimates across the different age groups (Figure 2) However, for the SA communities there was a downward trend in estimated ARTI with increasing age until the final (11 year old) cohort.

Using a linear regression model with ARTI as calculated by the fixed mirror method the slope of the line was −0.1% (95% CI:−0.3%, 0.1%; p = 0.3), indicating a small downward, but inconclusive, trend. Exploring the data further, and recognising the limitation of sub-group analyses, when we excluded 11 year-olds (as the smallest group) the slope was −0.2% (95% CI:−0.3%, −0.1%; p = 0.02), with clear evidence supporting a steeper downward trend. Broadly similar results were drawn when using ARTI estimates calculated from the other four methods.

### Effect of BCG

Scars typical of BCG vaccination were recorded in 74% of Zambian and 85% of SA children. Similar proportions of children within each country of both sexes (data not shown) and all ages had BCG scars.

**Figure 3:**
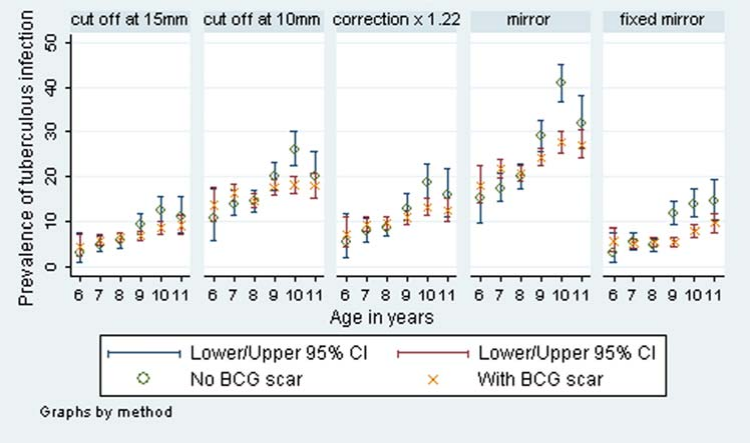
Prevalence of tuberculous infection with 95% binomial exact CIs, as calculated using five methods, by BCG and age for Zambia.

**Figure 4:**
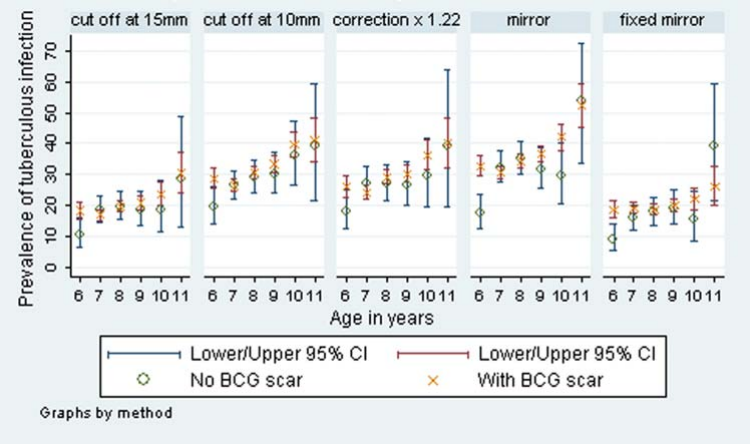
Prevalence of tuberculous infection with 95% binomial exact CIs, as calculated using five methods, by BCG and age for South Africa.

There was a 1–3% difference in infection prevalence estimates among children with a BCG scar and those without using country level estimates when different methods were compared (Table 3).The 10 mm cut off method showed the largest difference in infection prevalence estimates between children with a BCG scar and those without for SA whereas for Zambia and overall the largest respective difference was given by the mirror method. There was no convincing evidence of a greater effect in younger children in either country for any of the methods used (Figure 3 and Figure 4).

**Table 3:** Tuberculous infection prevalence estimates (95% confidence intervals) by country and overall.

	Zambia	Zambia	South Africa	South Africa	Overall*	Overall*
	*BCG present (N = 8,453)*	*BCG absent (N = 3,043)*	*BCG present (N = 6,745)*	*BCG absent (N = 1,181)*	*BCG present (N = 15,198)*	*BCG absent (N = 4,224)*
**15 mm**	7.4 (5.4–9.4)	7.3 (4.5–10.1)	19.6 (15.0–24.2)	17.7 (8.5–26.9)	11.5 (7.3–15.6)	10.8 (6.7–14.9)
**10 mm**	17.0 (12.0–22.0)	16.6 (8.9–24.4)	31.0 (22.8–39.2)	27.8 (11.0–44.7)	21.7 (15.0–28.3)	20.4 (13.1–27–7)
**14 mm** * **1.22**	11.2 (7.9–14.4)	10.9 (5.1–16.7)	28.6 (19.5–37.7)	25.7 (11.5–39.9)	17.0 (8.9–25.0)	15.8 (7.9–23.8)
**Mirror**	23.2 (14.5–31.9)	22.4 (10.9–33.9)	25.9 (0.7–51.1)	24.1 (0.0–49.9)	26.0 (19.1–32.9)	24.1 (16.3–32.0)
**Fixed mirror**	6.5 (4.0–9.1)	8.2 (0.3–16.2)	19.4 (12.3–26.6)	16.8 (0.0–42.0)	10.8 (5.8–15.8)	11.1 (3.7–18.5)

* 2,135 observations with unknown or doubtful BCG status

Country level and overall estimates are unweighted averages of community estimates and 95% Confidence intervals.

### Impact of different methods on community ranking in the ZAMSTAR study

Regardless of the approach used for the determination of tuberculous infection prevalence, there was little difference between the ZAMSTAR study intervention arms produced by the randomization procedures (Table 4). Absolute differences between approaches were present. To evaluate the association of community ranks by method, a correlation matrix of the ranks according to the five different methods was used (data not shown). Cut-off methods ranks tended to have very high correlation (r = 0.960 for ranks 10 mm vs. 15 mm; r = 0.987 for ranks 15 mm vs. 14 mm*1.22), whereas their comparison with mirror methods was not so (r = 0.681 for ranks 15 mm vs. mirror; r = 0.757 for ranks 10 mm vs. mirror).

**Table 4:** Average tuberculous infection prevalence estimates by intervention arm based on estimated prevalence calculated by various published methods.

ZAMSTAR Intervention arm [18]	15 mm	10 mm	14 mm*1.22	Mirror	Fixed mirror
Arm A	11.3	22.0	16.9	28.7 (n = 5)+	11.2
Arm B	11.4	20.5	16.8	25.8 (n = 5) +	11.6
Arm C	11.8	21.5	17.4	23.9	11.2
Arm D	10.5	20.7	15.7	23.7	10.1

+ These estimates are based on five and not six prevalence estimates as all other estimates in the table. This is because for two communities we could not clearly define a “true” mode of TST induration. Both these communities have low prevalence estimates as calculated by all other methods and would most probably produce low infection prevalence estimates for the mirror method too. We believe the observed differences in between intervention arms averages are an artefact of what we describe here.

## Discussion

This is the first time tuberculin survey data of this magnitude have been collected and presented from Zambia whereas, our data from Cape Town, largely agree with previous studies from the Western Cape of SA [28], [29]. These surveys have reported ARTI estimates ranging from 0.8% to 2.8% and 2.5% to 4.2% for Zambia and SA communities respectively, depending on the method we used. These ARTI estimates confirm that TB transmission is high in these communities, irrespective of the method used to define it. In addition, they remain comparatively higher than those reported in other African countries where TST surveys have been conducted [3], [4], [30] or worldwide [31]–[33]. Two recent TST surveys in Cape Town both showed an ARTI of 4.1% [28], [29] using the 10 mm cut off, largely agreeing with findings in this study.

Asymmetrical distributions, such as that seen in the Zambian communities have been reported from Tanzania [12] and are presumed to be due to a larger number of children being sensitised to environmental mycobacteria as found in places where the climate is more tropical. The mixture methods of analysis are designed to provide a better estimate of prevalence of infection in such situations than a simple cut-off based approach. A recent large prevalence survey of tuberculosis disease done in Zambia identified a large number of non-tuberculous mycobacteria [34].The rather symmetrical distribution seen in the cooler temperate areas of Cape Town has also been reported in other TST surveys [29]. In this situation, mixture methods are redundant since the distinction between infected and uninfected children is easily made and there are few children with intermediate results.

These TST surveys also confirm the difficulty in estimating absolute prevalence rates for tuberculous infection and highlight the danger in making comparisons across countries with different geographical and environmental conditions. The estimated absolute prevalence for each community or country varied widely. Our results are similar to other studies showing that estimates of infection prevalence vary widely depending on the method used to calculate them [10], [16]. Although various methodological approaches for the analysis of TST data have been suggested, more work on how to best interpret the results comparatively across different populations is needed.

The variability between populations in patterns of exposure and reactivity to different mycobacterial species means that judgement will remain an important element in the analysis and interpretation of such data [35].The conventional method of analyzing such data consists of presentation of tuberculin reaction sizes as a frequency distribution curve and locating an anti-mode on the curve, which is considered as the cut-off point for identification of the sub-group infected with *M.tuberculosis* [36]. However, a clear anti-mode is not always evident, especially in communities with high prevalence of cross-sensitivity to tuberculin. In such situations, mirror-image technique is used for estimating the prevalence of infection. However, identification of this mode poses further problems in communities with low prevalence of infection and high prevalence of cross-reactors [36]. Other statistical techniques for estimating the proportion of individuals infected with *M.tuberculosis* can then be employed such as mixture model analysis. The mixture model has been proposed as a possible solution to overcome problems in the interpretation of tuberculin surveys due to difficulties commonly encountered in the identification of modes and anti-modes of reactions due to infection with tubercle bacilli on the frequency distributions of reactions [37]. However, lack of supporting epidemiological evidence to show that mixture analysis is better than traditional methods has been raised by others [35].

When the communities are aggregated by country, the difference in tuberculous infection prevalence estimates between the Zambian and SA communities using mixture analysis is greater compared to other methods and the mode of the distribution of presumed infection is the same for both countries. Unfortunately, our non-zero induration data at the community level were not enough for conclusive results. As expected from the frequency distribution of induration in the SA children with very little cross-reaction, the mixture, the mirror and 10 mm cut-off methods give very similar results. Whereas in Zambia, with evident cross-reaction of environmental mycobacterial, the mixture method estimates are only similar to the 14 mm*1.22 method. Infection prevalence estimates obtained from mixture models have been shown to be lower [9], [32].or fairly concordant [9] with those from cut-off methods depending on the presence of environmental mycobacterial.

The mirror method shows the highest estimates of infection prevalence when countries are compared. When community level estimates are used, for both countries at the 15 mm cut-off point and at the 10 mm for the Zambian communities, the mirror method still showed higher estimates. At the 10 mm cut-off point, the SA communities show higher estimates by the mirror method in 50% of communities. The mirror method shows higher estimates than the 15 mm cut off method, for instance, since it counts as infected almost exactly twice the number of subjects when the assumed mode of the underlying distribution is 15 mm. In a study [15], using a range of chosen modes ,the mirror method yielded a wide variation in the estimates of the prevalence, a well-recognised problem. For the three cut off methods, infection prevalence was highest and lowest for the 10 mm and 15 mm cut off methods for each country. Several reasons have been raised for challenging the traditional cut-off approach to estimate infection prevalence [9].

The different methods did not greatly alter the ranking of the ZAMSTAR communities, and when used to stratify randomisation for the ZAMSTAR study, the infection prevalence estimates within each intervention arm differed very little, irrespective of method used. We have clearly demonstrated that the differences in method do not interfere with using any one method across 24 communities for randomization.

There was little difference among children that had BCG scar and those without, adding to the growing literature that when BCG is given at birth, little difference can be detected when using tuberculin skin test in children, adolescents or adults [22], [28], [38]. However, the 1–3% difference in infection prevalence estimates among children with a BCG scar and those without may be significant in countries with a low prevalence of tuberculous infection.

There is a lively debate about whether the rising incidence of active tuberculosis seen over the past decades in Southern Africa has led to higher risks of infection among children [39], [40]. It has been postulated that this rise in TB notification rates in adults is not well reflected by a corresponding increase in ARTI probably due to the less infectiousness of HIV positive individuals compared to HIV negative ones [40]. The youngest children in our surveys were exposed to tuberculosis from 1999, when they were born, until 2005 when the survey was performed. The oldest children were exposed from 1994 to 2005. Since the ARTI is calculated from the cumulative incidence of infection over the lifetime of the child, the differences in expected cumulative exposure are less dramatic than the changes in the incidence of tuberculosis. The WHO estimates that the incidence of tuberculosis reached a peak in Zambia in 2003, whereas it was still rising in South Africa in 2007 [41]. For the Zambian communities all methods agreed that the estimates of ARTI hardly vary for children of different ages, whereas there is a tendency for younger South African children to have higher estimates of ARTI. This is what would be expected if there was a direct relationship between cumulative incidence of tuberculosis and risk of infection. However, despite the much larger sample size in this study than the study that sparked the debate [29], we are not able to confirm or reject the hypothesis that 5-fold rise in tuberculosis rates in the whole of South Africa is reflected in higher infection risks for the younger cohorts in Cape Town.

Our study has some limitations. There is clear evidence of digit preference in the Zambian data despite efforts to standardise training and reading across the two country teams which may lead to under or overestimation of prevalence of infection. The use of six teams in Zambia may have also contributed to inter-reader variability. In addition, not all children vaccinated with BCG leave a scar and the scar may wane with time. Since the surveys were primarily conducted to obtain estimates to rank communities they were not necessarily a representative sample of the district or province from which they were drawn nor can the differences be extrapolated to compare Zambia and SA at the country level.

Although only 50% of registered children were injected and read, we believe that this has not lead to major bias in the interpretation of our results since there was no difference by age and sex among children registered, administered and read (Table 1). In Zambia, surveys of this nature were virtually unknown before ours and therefore our teams had initial difficulties in recruiting children which improved with increased community sensitization. Furthermore, since most large tuberculin surveys are often an integral part of the National Tuberculosis programs in countries with a high incidence of tuberculosis [24], [42], these do not require written consent, unlike ours. Parental written and informed consent often necessitates parents attending meetings where the study can be explained and this is difficult to achieve.

### Conclusion

Estimates of the annual risk of tuberculous infection are heavily dependent on the method used for calculating prevalence of infection and quantitative comparisons, particularly across large distances and different climate zones are likely to be flawed. Among the communities selected for the ZAMSTAR study, there are large variations in the prevalence of tuberculous infection, with substantially higher estimates in the SA communities than in the Zambian ones. TB transmission remains very high in these communities. We cannot exclude the possibility that the increasing tuberculosis notification rates, fuelled by the HIV epidemic in sub-Saharan Africa, have led to an increased risk of infection among school-children.

Our data add to the consensus that in settings where BCG is given at birth, results of tuberculin skin tests are not much affected by whether a child has been vaccinated or not. In this regard, the loss of specificity that is often cited as a reason to move to IGRAs [7] may be less important in typical African settings [43].
